# A Machine Learning Prediction Model for Non-cardiogenic Out-of-hospital Cardiac Arrest with Initial Non-shockable Rhythm

**DOI:** 10.14789/jmj.JMJ22-0035-OA

**Published:** 2023-05-20

**Authors:** SHINSUKE KARATSU, YOHEI HIRANO, YUTAKA KONDO, KEN OKAMOTO, HIROSHI TANAKA

**Affiliations:** 1 Department of Emergency and Critical Care Medicine, Juntendo University Urayasu Hospital, Chiba, Japan; 1 Department of Emergency and Critical Care Medicine, Juntendo University Urayasu Hospital, Chiba, Japan

**Keywords:** cardiac arrest, non-shockable rhythm, machine learning, prediction model, termination of resuscitation

## Abstract

**Objectives:**

The purpose of this study was to develop and validate a machine learning prediction model for the prognosis of non-cardiogenic out-of-hospital cardiac arrest (OHCA) with an initial non-shockable rhythm.

**Design:**

Data were obtained from a nationwide OHCA registry in Japan. Overall, 222,056 patients with OHCA and an initial non-shockable rhythm were identified from the registry in 2016 and 2017. Patients aged <18 years and OHCA caused by cardiogenic origin, cancer, and external factors were excluded. Finally, 58,854 participants were included.

**Methods:**

Patients were classified into the training dataset (n=29,304, data from 2016) and the test dataset (n=29,550, data from 2017). The training dataset was used to train and develop the machine learning model, and the test dataset was used for internal validation. We selected XGBoost as the machine learning classifier. The primary outcome was the poor prognosis defined as cerebral performance category of 3-5 at 1 month. Eleven prehospital variables were selected as outcome predictors.

**Results:**

In validation, the machine learning model predicted the primary outcome with an accuracy of 90.8% [95% confidence interval (CI): 90.5-91.2], a sensitivity of 91.4% [CI: 90.7-91.4], a specificity of 74.1% [CI: 69.2-78.6], and an area under the receiver operating characteristic value of 0.89 [0.87-0.92]. The important features for model development were the prehospital return of spontaneous circulation, prehospital adrenaline administration, and initial electrical rhythm.

**Conclusions:**

We developed a favorable machine learning model to predict the prognosis of non-cardiogenic OHCA with an initial non-shockable rhythm in the early stage of resuscitation.

## Introduction

A large number of out-of-hospital cardiac arrests (OHCAs) occur worldwide. The number of patients with OHCA in Europe and the United States is 275,000 and 42,000 per year^1)^, respectively. Emergency physicians and researchers have contributed a great deal of efforts and research to improve resuscitation. Unfortunately, the prognosis for patients with OHCA is still poor, especially for those with an initial non-shockable rhythm. In many cases, medical professionals must decide to stop resuscitation. However, it is difficult to accurately predict the prognosis for patients with OHCA at an early stage based on the scene of the emergency and the complex information it involves. The legitimacy of the decision to stop resuscitation is unclear, but the fact is that it is ultimately left to the individual judgment of medical professionals.

In recent years, the usefulness of prognostic models using machine learning has been reported on for patients with OHCA^2-4)^. Hirano et al. demonstrated the favorable performance of a machine learning model for predicting outcomes in patients with OHCA and an initial shockable rhythm^5)^. Reliable prognostication of this specific population with a high probability of cardiogenic cause might support clinicians' treatment choices, such as extracorporeal cardiopulmonary resuscitation, percutaneous coronary intervention, and temperature management. However, there has been no report of a machine learning model that can support clinician decision-making for discontinuing resuscitation specifically for patients with non-shockable rhythms, a population that has poor prognostic outcomes among patients with OHCA.

In this study, we aimed to develop and validate a machine learning model for patients with non- cardiogenic OHCA and non-shockable rhythms.

## Materials and Methods

### Study design, data sources, and ethical approval

This retrospective cohort study used data from the All-Japan Utstein Registry, a nationwide prospective OHCA registry established in 2005 by the Fire and Disaster Management Agency in Japan. The registry is based on a set of international Utstein-style guidelines and includes data on all patients with OHCA transported by emergency medical services in Japan. Survival and cerebral performance category (CPC) results 1 month after onset were included in this registry from 2016 to 2017.

The study protocol was approved by the Ethics Committee of Juntendo Urayasu Hospital (protocol number: U20-0011), and the requirement for informed consent was waived owing to the retrospective design.

### Study population and outcomes

**Figure 1:**
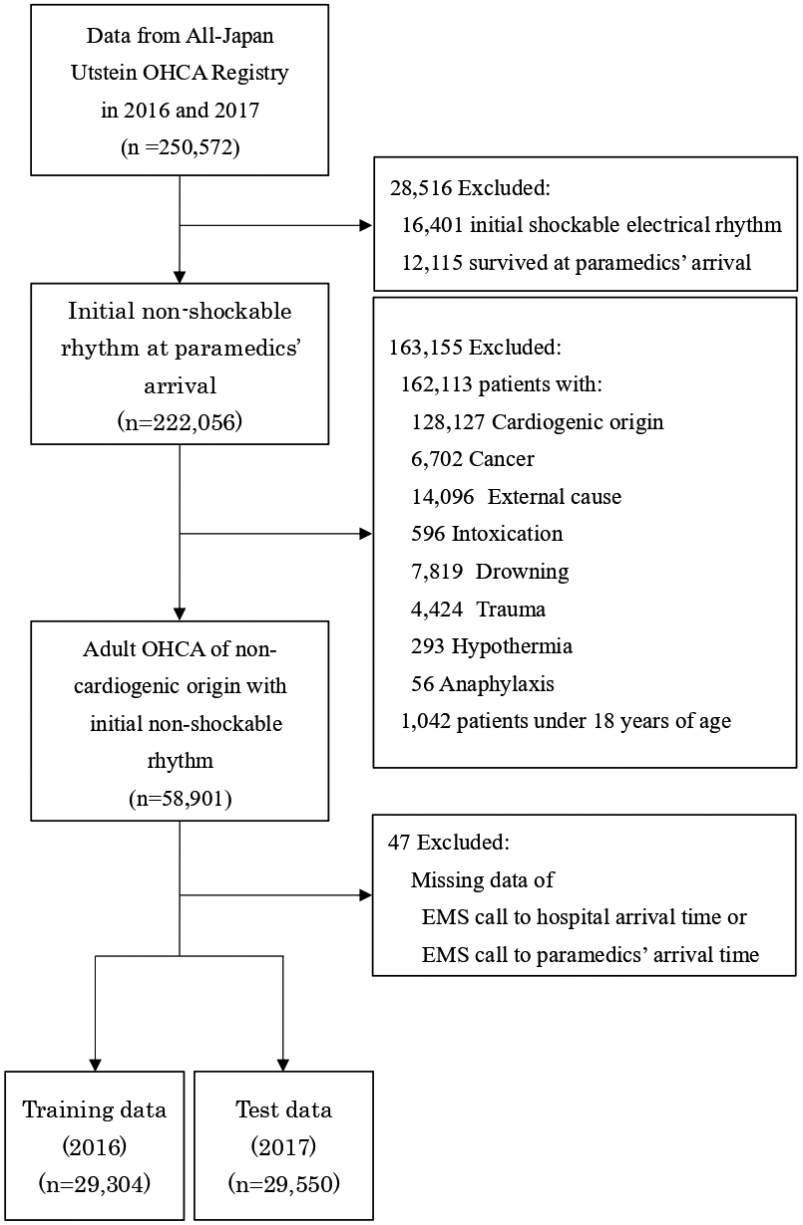
Flow diagram of patient inclusion OHCA: Out-of-hospital Cardiac Arrest, EMS: Emergency Medical Service

A flow diagram of the study population selection is shown in Figure 1. We extracted 250,572 patients with OHCA from the All-Japan Utstein Registry between 2016 and 2017. After we excluded 16,401 cases of initial shockable electrical rhythm and 12,115 patients who survived when paramedics arrived on-site, 222,056 patients with OHCA and an initial non-shockable rhythm were identified. Of these, patients aged <18 years and those with OHCA caused by cardiogenic origin, cancer, and external factors, including intoxication, trauma, accidental hypothermia, drowning, and anaphylaxis, were excluded, as they comprised separate patient subsets in terms of prognosis or cardiac arrest etiology. Finally, 58,901 adult patients with non-cardiogenic OHCA and an initial non-shockable rhythm were identified. After 47 cases with missing values for the minutes from the emergency medical service (EMS) call to hospital arrival time or EMS call to paramedics' site arrival time were deleted list-wise, data were subsequently classified into the training dataset (n=29,304, data from 2016) for the development of the machine learning model and the test dataset (n=29,550, data from 2017) for internal validation.

The primary outcome in this study was poor outcome at 1 month. A poor outcome was defined as CPC of 3-5. The secondary outcome was death at 1 month.

### Predictor Variables

Eleven prehospital variables were selected as outcome predictors of OHCA. These prehospital variables were sex, age, presence of prehospital physician, event witness, bystander cardiopulmonary resuscitation (CPR), initial electrical rhythm, prehospital defibrillation, prehospital adrenaline administration, return of spontaneous circulation (ROSC), EMS call to hospital arrival time, and EMS call to paramedics' site arrival time.

### Training of the machine learning model

Using the training dataset, we trained and developed an XGBoost machine learning model as a classifier for outcome prediction. In the training process, a 10-fold cross-validation was performed. The training data were split into 10 stratified subsets. Nine subsets (90% of training data) were used to train the model, and the remaining subset (10% of training data) was used for validation. These training and validation processes were repeated 10 times with each subset used once as a validation dataset, allowing us to obtain 10 estimates of predictive accuracy, which were averaged to obtain a single estimate. Thus, we avoided overfitting the model and searched for hyperparameters to obtain the best accuracy for outcome prediction. The weighting of rare outcomes by the ratio of the number of minor classes to the majority class was also used to control the outcome imbalance of the data.

### Internal validation of the machine learning model

The performance of the developed machine learning model was validated using the test dataset. We measured the area under the receiver operating characteristic curve (AUROC), area under the precision-recall curve (AUPR), sensitivity, specificity, positive predictive value (PPV), negative predictive value (NPV), and accuracy as performance indicators. The feature importance for developing the XGBoost model was computed as the normalized total reduction of the criterion brought about by the feature, which is known as the Gini importance.

### Statistical analysis and library for machine learning

Scikit-learn (version 0.21.3) with Python was employed for model development. Statistical analyses of the characteristics of the cohorts were performed using SciPy (version 1.4.1) and Python (version 3.7.4, in Anaconda 2019.10). Continuous variables are reported as means and standard deviations, and categorical variables are reported as counts and percentages. A t-test was used to compare the means between the two samples. A chi-square test was used to compare the frequencies. All tests were two-sided, and the significance level was set at 5% (p<0.05).

## Results

### Characteristics of study participants

The main characteristics of included patients with non-cardiogenic OHCA and an initial non- shockable rhythm are shown in Table 1. The mean age of the patients was 77.2 years, and 53.6% were men. The initial electrical rhythm comprised of 30.8% pulseless electrical activity and 69.2% asystole. Event witness and bystander CPR were observed in 46.1% and 25.0% of all cases, respectively. When comparing the training and test datasets, significantly lower rates of bystander-performed CPR were observed in the test dataset than those in the training dataset (23.6% vs. 26.3%, respectively). In contrast, adrenaline was administered more frequently at the prehospital scene in the test dataset (22.6%) than that in the training dataset (20.9%). The minutes from the EMS call to paramedics site arrival time was statistically different between these two cohorts; however, the absolute value of the difference was quite low.

**Table 1:** Characteristics of study participants

Variable	All (n=58,854)	Training data (n=29,304)	Test data (n=29,550)	P value
Age (years)	77.2 [14.8]	77.1 [14.9]	77.4 [14.7]	0.27
Sex (men)	31,544 (53.6%)	15,699 (53.5%)	15,845 (53.6%)	0.91
Event witness	27,150 (46.1%)	13,567 (46.2%)	13,583 (46.0%)	0.42
Bystander CPR	14,690 (25.0%)	7,730 (26.3%)	6,960 (23.6%)	<0.01
Initial electrical rhythm				0.76
Pulseless electrical activity	18,154 (30.8%)	9,056 (30.9%)	9,098 (30.8%)	
Asystole	40,700 (69.2%)	20,248 (69.0%)	20,452 (69.2%)	
Defibrillation	1,671 (2.8%)	849 (2.9%)	822 (2.8%)	0.47
Prehospital ROSC	6,400 (10.9%)	3,137 (10.7%)	3,263 (11.0%)	0.19
Prehospital physician	2,029 (3.4%)	1,046 (3.5%)	983 (3.3%)	0.11
Adrenaline administration	12,816 (21.8%)	6,126 (20.9%)	6,690 (22.6%)	<0.01
EMS call to hospital arrival time (minutes)	33.9 [13.0]	33.9 [13.3]	33.9 [12.7]	0.62
EMS call to paramedics' site arrival time (minutes)	9.2 [4.4]	9.1 [4.6]	9.2 [4.2]	0.01
Outcomes				
Poor outcome at 1 month	58,142 (98.8%)	28,943 (98.8%)	29,199 (98.8%)	0.62
Death at 1 month	56,259 (95.6%)	28,006 (95.6%)	28,253 (95.6%)	0.81

Categorical variables are presented as n (%). Continuous variables are presented as the mean [standard deviation]. CPR, cardiopulmonary resuscitation; ROSC, return of spontaneous circulation; EMS, emergency medical service.

### Performance of the developed machine learning model

**Figure 2:**
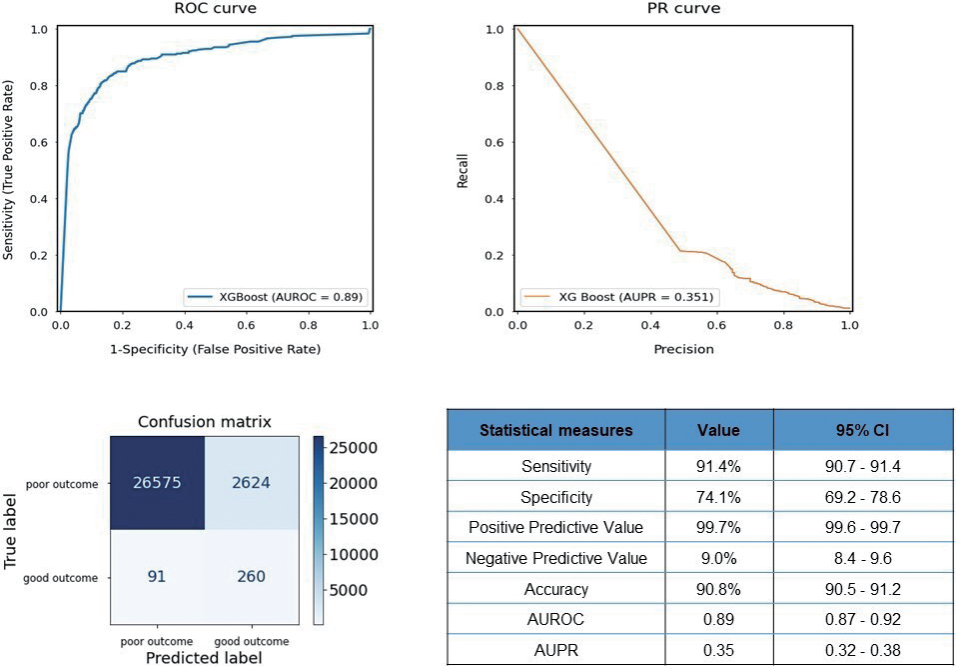
ROC curve, confusion matrix, and statistical measures of performance of the machine learning model to predict primary outcome AUROC: Area Under the Receiver Operating Characteristic Curve, AUPR: Area Under the Precision Recall, CI: Confidence Interval.

Figure 2 shows the ROC curve; PR curve; confusion matrix; and evaluation measures such as sensitivity, specificity, PPV, NPV, accuracy, AUROC, and AUPR values obtained in the test set model validation for the primary outcome. For the prediction of death or survival with poor neurological function at 1 month, the developed machine learning model demonstrated a favorably high AUROC value of 0.89 (95% confidence interval [CI]: 0.87-0.92). In contrast, the AUPR was relatively low at 0.35 (95% CI: 0.32-0.38). The sensitivity and specificity were 91.4% and 74.1%, respectively. It also showed a high PPV (99.7%). The accuracy of the validation was 90.8%.

**Figure 3:**
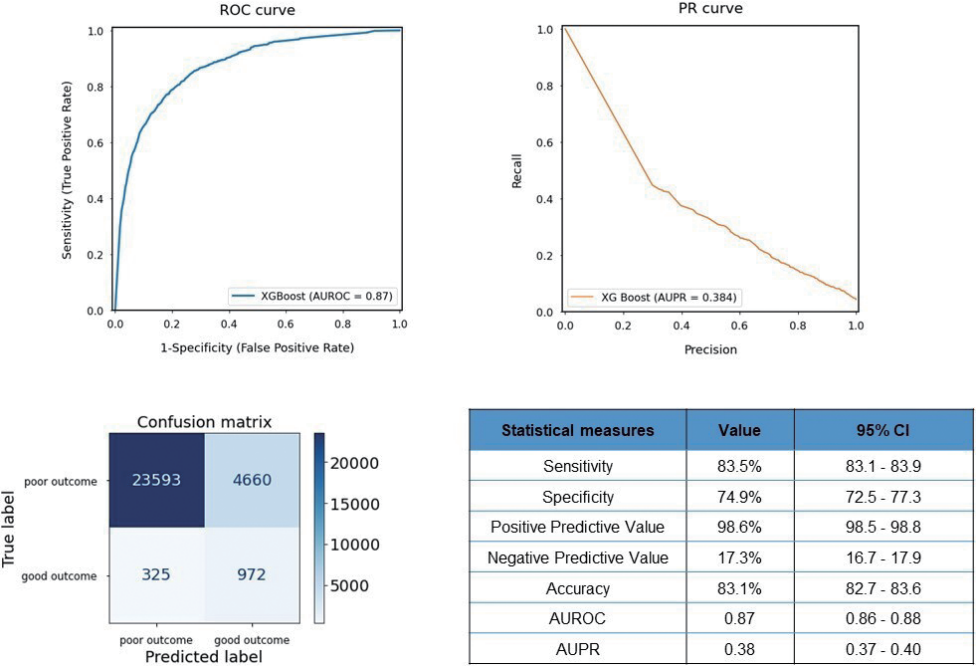
ROC curve, confusion matrix, and statistical measures of performance of the machine learning model to predict secondary outcome AUROC: Area Under the Receiver Operating Characteristic Curve, AUPR: Area Under the Precision Recall, CI: Confidence Interval.

Figure 3 shows the ROC curve; PR curve; confusion matrix; and evaluation measures such as sensitivity, specificity, PPV, NPV, accuracy, AUROC, and AUPR values obtained in the test set model validation for the secondary outcome. For the prediction of death at 1 month, the developed machine learning model demonstrated a favorably high AUROC value of 0.87 (95% CI: 0.86-0.88). In contrast, the AUPR was relatively low at 0.38 (95% CI: 0.37-0.40). The sensitivity and specificity were 83.5% and 74.9%, respectively. It also showed a high PPV (98.6%). The accuracy of the validation was 83.1%.

### Evaluation of feature importance

**Figure 4:**
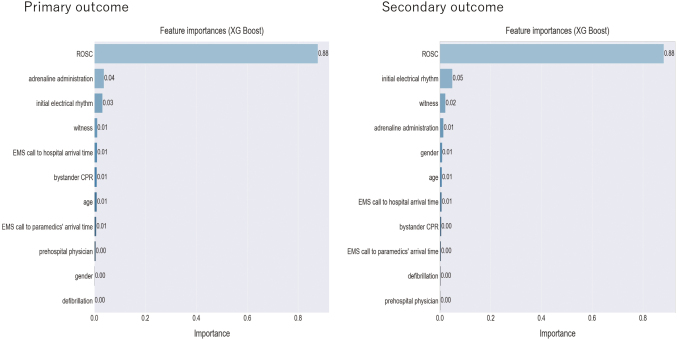
Feature importance of the model variables ROSC: Return of Spontaneous Circulation, EMS: Emergency Medical Service, CPR: Cardiopulmonary Resuscitation.

Figure 4 shows the feature importance for developing the machine learning model. The essential feature to develop the model was the prehospital ROSC. The second and third most important features were adrenaline administration and initial rhythm for the primary outcome, and initial rhythm and event witness for the secondary outcome. However, they were much less decisive features than the prehospital ROSC.

## Discussion

To the best of our knowledge, this study is the first to develop and internally validate a machine learning-based outcome prediction model targeting the population of patients with non-cardiogenic OHCA and an initial non-shockable rhythm. Our purpose was to assess the predictive performance of the machine learning in OHCA and its possibility of use in the clinical setting in the future. In summary, our machine learning model, developed using 11 prehospital variables from the All-Japan Utstein Registry, showed a favorable prognostic performance in predicting a poor outcome of OHCA at 1 month, with a high PPV of 98.6%, encouraging the possibility of the model being used to decide the termination of resuscitation for patients with non- cardiogenic OHCA and a non-shockable rhythm.

Individuals under 18 years of age and those with OHCA caused by cardiogenic origin, cancer, and external factors were excluded from the study population. This selection of the OHCA population for the study was considered in order to include adult patients with endogenous cardiac arrest, whose cases often involve an uncertainty about the decision to interrupt resuscitation in the emergency department. In addition, this study did not include patients who were not in cardiac arrest at the time of EMS contact, even if they experienced cardiac arrest with an initial non-shockable rhythm. These patients were not included because they had a high chance of being resuscitated; thus, clinicians were not deciding on the early termination of resuscitation (TOR). Thus, we carefully selected patients for inclusion in the study in view of the usability of the prediction model to determine TOR in clinical practice.

Many efforts have been made to create specific rules for the TOR without relying solely on the clinician's judgment. Various TOR rules have been developed and validated^6-10)^. A very high PPV is required for the use of TOR rules, owing to their ethical aspects. Similar to other TOR rules, our machine learning model showed a very high PPV of 99.7% in the internal validation. Although the question remains whether this value of 99.7% is sufficient for resuscitation interruption, it is considered reasonable to judge futility based on a percentage of expected therapeutic effect of 1% or less in Europe and the United States^11)^. Therefore, the results of our study may provide a basis for using this machine learning prediction model for clinical use. However, it is necessary to consider not only whether the patient is alive or dead but also whether the family is present when resuscitation is interrupted and other aspects involving the time of death diagnosis. Ultimately, TOR rules should be carefully introduced into the emergency medical system based on the public's ethical viewpoints.

The feature with the greatest importance for our machine learning model development in the primary and secondary outcome was ROSC. Unfortunately, the results showed that OHCA cases with initial non-shockable rhythms that were not successfully resuscitated in prehospital settings resulted in poor outcomes. The second contributing feature in the primary outcome was prehospital adrenaline administration, although it made a very small contribution to the development of the prognostic model compared with the feature of prehospital ROSC. Although none of the previous TOR rules included prehospital adrenaline administration, early adrenaline administration in patients with OHCA and a non-shockable rhythm has been reported to increase ROSC rates^11-14)^. Therefore, early administration of adrenaline is likely to be an important prognostic factor. Similarly, in the secondary outcome, initial electrical rhythm and witness were related to poor outcomes following ROSC. Thus, these prognostic features derived from our machine leaning models are consistent with historically proven, general understanding of clinicians and researchers that no prehospital ROSC, no prehospital adrenaline administration, no witnesses, and a non-shockable rhythm on the initial electrical rhythm are associated with a poor prognosis

Our machine learning model requires 11 predictive variables, more than the number of other TOR rules. Other TOR rules have only three to five criteria; therefore, it is easier to decide the discontinuation of resuscitation in the field because of simplicity. However, it is possible for our model to overcome this limitation by using technologies such as speech recognition or optical character recognition.

Our study has a strength in the use of a nationwide database. Previous studies on OHCAs used datasets restricted to specific regional areas^15, 16)^, and access to medical facilities, population density, and patient characteristics (such as underlying diseases) may differ between urban and rural areas. This difference may have a strong influence on the outcome of patients with OHCA. The nationwide Utstein database used in this study eliminates these regional differences. Additionally, another strength of our study is not only to review the importance of poor outcome prognostic factors have been reported using machine learning but also to indicate a possibility that we can put the result to clinical use using some devices such as applications.

Our study had several limitations. First, this study was based only on data from Japanese patients for model training and validation. Therefore, the results cannot be generalized to countries with different emergency care systems. External validation using datasets from other communities or countries is also required. Second, listwise deletion of cases with missing data was performed during the data-cleaning process, which can decrease the sample size and cause bias in the parameter estimates. However, other methods to deal with missing data, such as multiple imputations, also cause bias. Moreover, some studies have used the same database and also treated missing data with listwise deletion^17)^. Third, the dataset used for the current study was a bit old, precluding the guarantee of similar performance in future cases. However, the sample size is large, and remarkable innovation in the diagnostic or treatment process of OHCA resuscitation has not occurred in recent years. Thus, there is no substantial reason that influences model performance. Nevertheless, it is better that the machine learning model should be hopefully re-assessed and re-validated using the new dataset. Especially, when the model is intended to use in the clinical setting, the model should be validated using the new and external dataset.

In conclusion, we developed a favorable machine learning model to predict the prognosis of non-cardiogenic OHCA with an initial non-shockable rhythm using only prehospital information. Although the model should be externally validated in the future, this study has demonstrated the potential of a machine learning-based outcome prediction model in facilitating TOR decision-making for non-cardiogenic OHCA with an initial non-shockable rhythm.

## Funding

No funding was received.

## Author contributions

SK and YH analyzed and interpreted the patient data regarding out-of-hospital cardiac arrest with an initial non-shockable rhythm. All authors have read and approved the final manuscript.

## Conflicts of interest statement

YH is the Chief Executive Officer, MedPop Co. Ltd. None of the other authors declare no conflict of interest.
